# A mechanism of resistance to TRAIL/Apo2L-induced apoptosis of newly established glioma cell line and sensitisation to TRAIL by genotoxic agents

**DOI:** 10.1038/sj.bjc.6600666

**Published:** 2003-01-28

**Authors:** Y Arizono, H Yoshikawa, H Naganuma, Y Hamada, Y Nakajima, K Tasaka

**Affiliations:** 1 Department of Orthopedic Surgery, Yamanashi Medical University, 1110 Shimokato, Tamaho-cho, Yamanashi 409-3898, Japan; 2 Department of Parasitology & Immunology, Yamanashi Medical University, 1110 Shimokato, Tamaho-cho, Yamanashi 409-3898, Japan; 3 Department of Neurosurgery, Yamanashi Medical University, 1110 Shimokato, Tamaho-cho, Yamanashi 409-3898, Japan

**Keywords:** TRAIL, TRAIL receptors, apoptosis, p53, genotoxic agents

## Abstract

Most tumour cells are sensitive to TRAIL-induced apoptosis, but not normal cells; thus, cancer therapy using TRAIL is expected clinically. Several tumour cells are resistant to TRAIL-induced apoptosis, and various mechanisms of such resistance were reported in individual cases. In this study, we established a TRAIL-resistant glioma cell line, which completely lacked TRAIL receptors. In addition, this tumour cell line had wild-type p53 tumour-suppressive gene, suggesting new mechanisms for tumour cells to expand and escape from immune surveillance. The present study further explored the mechanisms that determine the sensitivity to TRAIL. We show that genotoxic agents such as cisplatin, doxorubicin and camptothecin, in addition to UV radiation, can induce TRAIL-R2 on the cell surface of TRAIL receptor-negative tumour cells. Newly synthesised TRAIL-R2 is functional, so apoptosis is effectively induced by TRAIL, but it is significantly inhibited by constitutive expression of dominant-negative p53. In addition, apoptosis induced by pretreatment of genotoxic agents and additional stimulation of TRAIL is efficiently inhibited by either antagonistic anti-TRAIL-R2 antibody or pan-caspase inhibitor z-VAD-FMK. Taken together, these findings suggest that resistance to TRAIL by lack of TRAIL receptors on glioma is restored by genotoxic agents, which support the new strategies for tumour killing by TRAIL-bearing cytotoxic cells in combination with genotoxic treatment.

TRAIL/Apo2L, a cytotoxic ligand that is a member of the TNF family (Wiley *et al*, 1995; Pitti *et al*, 1996), and its specific receptors (Degli-Esposti *et al*, 1997a; MacFarlane *et al*, 1997; Marsters *et al*, 1997; Pan *et al*, 1997a,1997b; Screaton *et al*, 1997; Sheridan *et al*, 1997; Schneider *et al*, 1997; Ashkenazi and Dixit, 1999a) have been identified. Similar to other TNF family molecules such as TNF-*α*, FasL or TWEAK, TRAIL can induce apoptosis by ligation to its specific cell surface receptors (TRAIL-Rs). Although TRAIL can induce apoptosis in transformed cells of diverse origin, little or no effect on normal cells has been reported (Zhang *et al*, 2000). In contrast to FasL, the administration of which causes lethal liver failure in mice (Tanaka *et al*, 1997), injection of TRAIL was suggested not to reveal general toxicity (Ashkenazi *et al*, 1999b; Walczak *et al*, 1999), although it was recently shown to induce damage in human hepatocytes and normal brain tissues (Jo *et al*, 2000; Nitsch *et al*, 2000).

The regulation of TRAIL-induced apoptosis was suggested to be mostly controlled by the expression of TRAIL receptors. Among TRAIL receptors, two distinct receptors for TRAIL, TRAIL-R1 (DR4) and TRAIL-R2 (DR5), which have intracellular death domains, have been identified. Two other receptors for TRAIL, TRAIL-R3 (DcR1), which has no cytoplasmic death domain, and TRAIL-R4 (DcR2), which has an incomplete death domain, have also been identified. TRAIL-R3 and -R4 were suggested to act as decoy receptors and to inhibit TRAIL-induced apoptosis by competition with TRAIL-R1 and -R2. Although the expression of TRAIL-R1 or -R2 is necessary to induce the death signal of TRAIL, the expression of TRAIL-R3 and -R4 does not necessarily correlate with cell sensitivity to TRAIL (Griffith *et al*, 1998; Hao *et al*, 2001). Recently, mutations of TRAIL-R1 and -R2 have been reported as the mechanism for tumour cells to be resistant to TRAIL in breast cancers (Shin *et al*, 2001). On the contrary, the expression of intracellular inhibitors of apoptosis has been proposed to be an essential determinant of cell sensitivity to TRAIL. The expression level of FLICE-inhibitory protein (FLIP) was higher in the TRAIL-resistant cells than TRAIL-sensitive cells (Griffith *et al*, 1998). Downregulation of caspase-8 was also reported in TRAIL-resistant Ewing's sarcoma cells (Kontny *et al*, 2001). Correlation between PED/PEA-15 protein or erbB-2 receptor and the TRAIL resistance was reported in glioma and other cell lines (Cuello *et al*, 2001; Hao *et al*, 2001).

To overcome the resistance of tumour cells to TRAIL, enhanced effects using combinations of anticancer drugs and TRAIL have been reported (Lacour *et al*, 2001). Among them, several anticancer drugs upregulate the expression of TRAIL-R1 or -R2 by both p-53-dependent and -independent mechanisms (Sheikh *et al*, 1998). However, the effects of each drug and the mechanisms of individual cases to enhance the expression of TRAIL receptors are complicated. In a present study, we established a TRAIL-resistant glioma cell line that completely lacked the expression of TRAIL receptors but had wild-type p53, and we examined the mechanisms for sensitisation to TRAIL-induced apoptosis by genotoxic agents or UV radiation using a TRAIL-receptor-negative cell line.

## Materials and Methods

### Reagents and antibodies

Soluble human TRAIL (Apo2L) was purchased from BIOMOL (Plymouth Meeting, PA, USA). Cisplatin was purchased from Sigma (St. Louis, MO, USA), and doxorubicin and camptothecin were purchased from TopoGEN (Columbus, OH, USA). A goat anti-human TRAIL-R2 Ab was purchased from DAKO (Kyoto, Japan). Normal goat IgG was purchased from ZYMED (So. San Francisco, CA, USA), and FITC-conjugated donkey anti-goat IgG Ab was purchased from BETHYL (Montgomery, TX, USA). z-VAD-FMK was purchased from PEPTIDE INSTITUTE (Osaka, Japan).

### Cell culture

Human osteosarcoma cell lines, HOS, MG63, NY, SaOS, human glioma cell lines, T98G, A172, KG-1-C and fibrosarcoma HT1080 were maintained in RPMI 1640 medium (GIBCO, Grand Island, NY, USA) supplemented with 10% heat-inactivated FCS (Egquitech-Bio, Ingram, TX, USA), 2 mM L-glutamine, 1×10^−5^ M β-mercaptoethanol and 0.1 mg/ml of kanamycin at 37°C in a humidified 5% CO_2_ atmosphere. Human normal osteoblasts were purchased from Sanko-junyaku (Tokyo, Japan) and maintained with Osteoblast Growth Medium (Osteoblast Growth Medium Bullet Kit). Human normal fibroblasts were obtained in our laboratory from a single donor who underwent an operation for osteoarthritis. The #63 glioma cell line was also established in our laboratory from a patient with glioma. Briefly, tumour cells obtained by operation were cultured in a flask. The #63 cells used in this study were cultured *in vitro* repeatedly more than 63 times generation after generation but were not cloned.

### Measurement of cell viability

We measured cell viability using a crystal violet assay. Briefly, 2×10^4^ cells were cultured in the presence of TRAIL with or without cisplatin, doxorubicin or camptothecin in a 96-well microtiter plate. In each assay, z-VAD-FMK or anti-TRAIL-R2 Ab was added 1 h before the addition of TRAIL. After removal of the medium, cells were stained with 50 *μ*l crystal violet solution (0.5%) for 5 min. Then cells were rinsed 10 times in tap water and the plates were dried on paper. After 1 h, 100 *μ*l of equal volume mixtures of ethanol and 0.1 M sodium phosphate were added and the absorbance at 570 nm was measured using a Titertek Multiscan Plus microplate reader (Flow Laboratories Inc., McLean, VA, USA).

### DNA fragmentation assay

The #63 cells were incubated with or without 2.5 *μ*M of camptothecin for 24 h and further cultured in the presence or absence of TRAIL for 24 h. Then 2×10^6^ cells were washed with phosphate-buffered saline (PBS), lysed in 30 *μ*l of 10 mM Tris-HCl, 100 mM EDTA-Na (pH 8.0), containing 0.5% (w/v) sodium-*N*-lauryl sulphate (SDS) and 0.5 mg/ml RNase A (Sigma, St Louis, MO, USA), and incubated at 50°C for 30 min followed by treatment with 0.5 mg/ml of proteinase K (Sigma, St. Louis, MO, USA) at 50°C for 30 min. Electrophoresis was performed on 2% agarose gel and DNA was visualised by ethidium bromide staining.

### Flow cytometric analysis

The cell surface TRAIL-R2 expression was measured using flow cytometric analysis. Briefly, a total of 1×10^6^ cells were incubated with or without each agent in a 24-well plate. Then, anti-TRAIL-R2 Ab or normal goat IgG was added and incubated for 1 h on ice. After washing with PBS, cells were incubated with FITC-conjugated donkey anti-goat IgG Ab. Flow cytometric analyses were performed using a FACSCalibur flow cytometer (Becton Dickinson, Mountain View, CA, USA).

### RT-PCR

Total RNA was isolated using the guanidium isothiocyanate method from the tumour tissue or cells cultured with or without each agent. Total RNA (5 *μ*g) was reverse transcribed with MuLV reverse transcriptase. Reverse transcription was performed using a thermal programme of 42°C for 60 min and 90°C for 5 min. The PCR reaction was performed using the following primers: TRAIL-R1, 5′-CTG AGC AAC GCA GAC TCG CTG TCC AC-3′ and 5′-TCC AAG GAC ACG GCA GAG CCT GTG CCAT-3′; TRAIL-R2, 5′-GCC TCA TGG ACA ATG AGA TAA AGG TGG CT-3′and 5′-CCA AAT CTC AAA GTA CGC ACA AAC GG-3′; TRAIL-R3, 5′-GAA GAA TTT GGT GCC AAT GCC ACT G-3′ and 5′-CTC TTG GAC TTG GCT GGG AGA TGT G-3′; TRAIL-R4, 5′-CTT TTC CGG CGG CGT TCA TGT CCT TC-3′ and 5′-GTT TCT TCC AGG CTG CTT CCC TTT GTA G-3′. The thermal programme was one cycle at 94°C for 5 min, 30 cycles of 94°C for 1 min, 55°C for 1 min, 72°C for 2 min, and 5 min at 72°C. PCR-amplified products were resolved and electrophoresed on 2% agarose gel and visualised with ethidium bromide.

### Transfection of the dominant-negative p53 gene into #63 cells

The construct of the dominant-negative p53 (DN-p53) gene was designed as previously described (Curtin *et al*, 2001) and was donated by Dr Spinella (Dartmouth Medical School, Hanover, NH). DN-p53 containing plasmid and control vehicle (pcDNA3.1MycHisA, Invitrogen, Cavlsbad, CA, USA) was individually transfected into #63 cells using Effectene Transfection Regent (Qiagen, Hilden, Germany). After 24 h culture, cells were incubated with the optimal concentration of G-418 and successful transfectants were cloned after 2 weeks culture.

### Western blotting

Proteins (5 *μ*g) obtained by SDS lysis were separated on 12.5% polyacrylamide gel. After transfer to a polyvinylidene difluoride membrane and incubation overnight at 4°C with 5% bovine serum albumin (BSA) in PBS to block nonspecific immunoglobulins, the membrane was incubated for 1 h at room temperature with anti-p53 monoclonal Ab (Oncogene, Boston, MA, USA). After washing, the membrane was incubated with HRP-conjugated secondary Ab for 1 h at room temperature, and specific bands were detected using ECL, according to the manufacturer's protocol (Amersham, Arlington Heights, IL, USA).

### Statistics

The statistical significance was analysed using Student's *t*-test. Values are presented means±s.d. Differences were considered to be significant when *P*<0.05.

## Results

### TRAIL induces apoptosis of human osteosarcoma, fibrosarcoma and glioma cells but not normal cells

**Figure 1:**
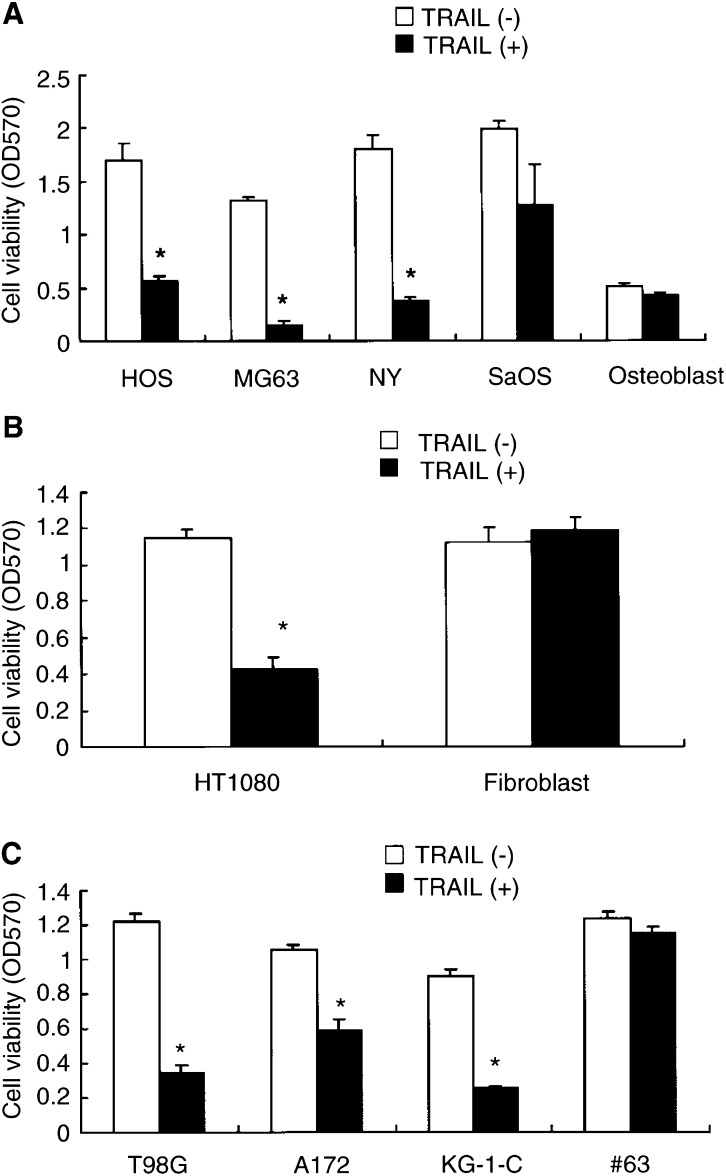
Sensitivity of osteosarcoma, fibrosarcoma, glioma cell lines, human normal osteoblasts and fibroblasts to TRAIL. (**A**) After 48 h of culture with 500 ngml^−1^ of TRAIL, the cell viability of HOS, MG63, NY, SaOS and osteoblasts was determined by crystal violet assay as described in Materials and Methods. The means and standard deviations of three independent experiments are shown. Statistical significance: ^*^P<0.01 compared with controls. (**B**) After 48 h of culture with 2 *μ*gml^−1^ of TRAIL, the cell viability of HT1080 and fibroblasts was determined by crystal violet assay. The means and s.d.'s of five independent experiments are shown. Statistical significance: ^*^P<0.01 compared with controls. (**C**) After 48 h of culture with 2 *μ*gml^−1^ of TRAIL, the cell viability of T98G, A172, KG-1-C, and #63 was determined by crystal violet assay. The means and s.d.'s of five independent experiments are shown. Statistical significance: ^*^P<0.01 compared with controls.

**Figure 2:**
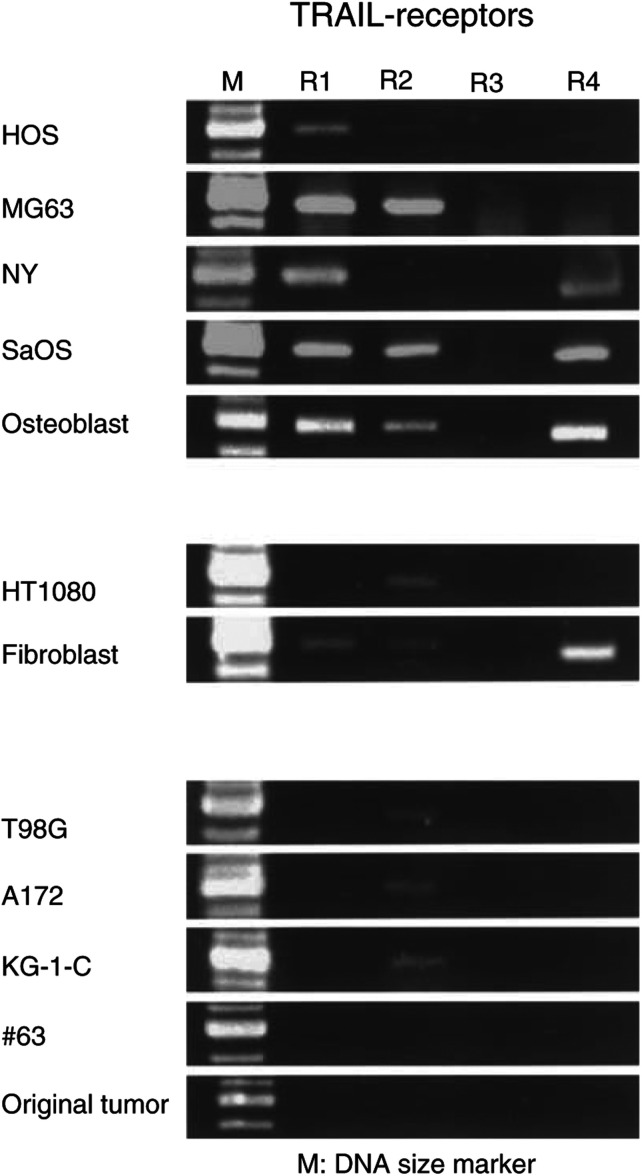
Expression of TRAIL receptors mRNA. RT-PCR was performed using specific primers to each TRAIL receptor. Total RNA (5 *μ*g) was reverse transcribed and PCR amplified. Each product was electrophoresed on 2% agarose gel and was visualised with ethidium bromide. The length of each PCR product matched that predicted from the sequence data.

We first examined the cytotoxic effects of TRAIL against some tumour cell lines, including the newly established tumour cell lines in our laboratory and normal cells using crystal violet assay as described in Materials and Methods. As shown in Figure 1A–C

, cell death was induced by exposure to TRAIL for 48 h in all tumour cells except #63, but not in normal osteoblasts or fibroblasts. Among them, however, #63 glioma cells were resistant to TRAIL. It was reported that some tumour cells become resistant to TRAIL-induced cytotoxicity in various ways. To investigate the mechanism for TRAIL resistance of #63 glioma cells, we first performed RT-PCR to detect the expression of TRAIL receptors. Either TRAIL-R1 (DR4) or TRAIL-R2 (DR5), which is necessary to induce a death signal by TRAIL, was detectable in any cell line except #63 glioma cells (Figure 2

). TRAIL-R3 (DcR1) was not detectable in any cell line and TRAIL-R4 (DcR2) was only detected in osteosarcoma cell lines, such as HOS, NY, SaOS, and normal osteoblasts and fibroblasts. The expression level of DcR2 had a tendency to correlate with the sensitivity of TRAIL-induced cell death. However, #63 glioma cells did not express TRAIL receptors at all. To deny the possibility that the lack of TRAIL receptors of #63 cells occurred *in vitro* during repeated culture, we examined the expression of TRAIL receptors of original tumour tissue. As shown in Figure 2 (lower panel), TRAIL receptors were not expressed in the original tumour tissue, suggesting an important mechanism for resistance of tumour cells to TRAIL *in vivo*. Many tumour cells have been reported to have mutations in the p53 tumour-suppressive gene and it is frequently one possible mechanism for progression of tumours. Therefore, we investigated the sequence of p53 gene of #63 cells and confirmed that they had wild-type p53 (data not shown). Together, #63 cells are unique tumour cells that completely lack the expression of TRAIL receptors and have wild-type p53, suggesting a unique mechanism for the expansion of tumour cells.

### A combination of TRAIL and genotoxic agents induces synergistic cytotoxicity in #63 glioma cells

**Figure 3:**
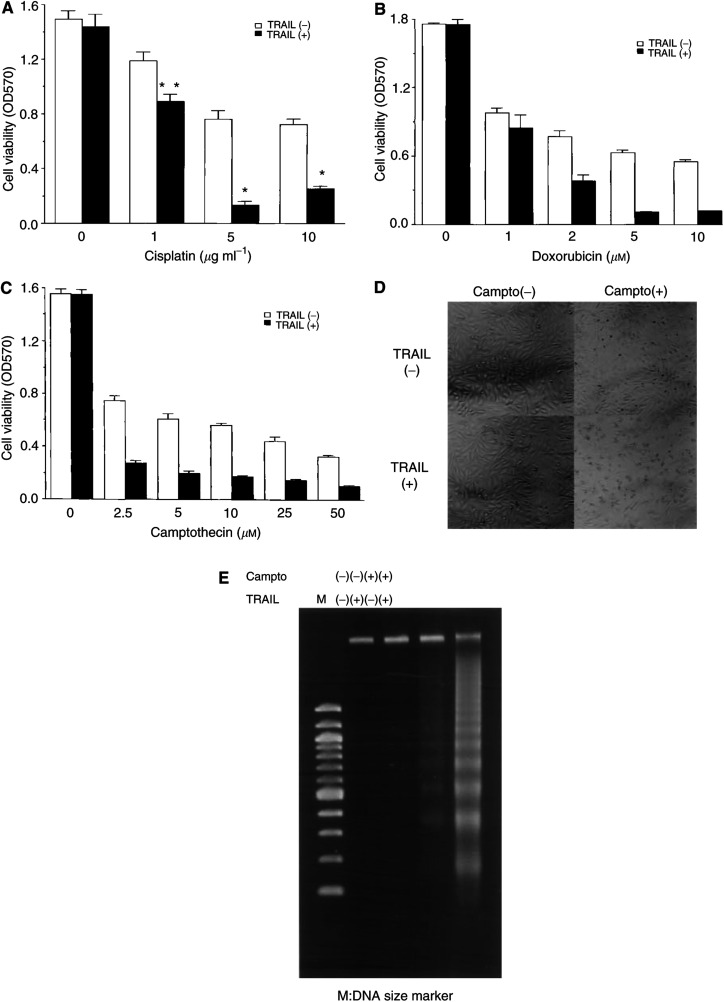
Cell viability and apoptotic changes in #63 cells treated with combinations of TRAIL and genotoxic agents. The #63 cells were cultured with various concentrations of cisplatin (**A**), doxorubicin (**B**) and camptothecin (**C**) for 24 h and further cultured with or without TRAIL (2 *μ*gml^−1^) for 48 h. Cell viability was determined by crystal violet assay. The means and s.d.'s of five independent experiments are shown. Statistical significance: ^*^P<0.01, ^**^P<0.05 compared with controls. The #63 cells were cultured with or without camptothecin (2.5 *μ*M) and they were further cultured in the presence or absence of TRAIL (2 *μ*gml^−1^). Morphological changes were observed under a phase-contrast microscope (×100) (**D**) and DNA fragmentation assay was performed as described in Materials and Methods (**E**).

Recently, the enhanced cytotoxity of anticancer drugs and TRAIL was reported in several cancer cells. Therefore, we next studied whether a combination of TRAIL with genotoxic agents could induce cytotoxicity in #63 glioma cells, which were resistant to TRAIL-induced cell death. We measured the viability of #63 glioma cells upon exposure to TRAIL with a wide range of concentrations of cisplatin, doxorubicin or camptothecin using crystal violet assay. As shown in Figure 3A

, cisplatin itself induced the growth inhibition of #63 glioma cells at concentrations higher than 5 *μ*gml^−1^, and it was significantly enhanced in the presence of TRAIL. As in the case of cisplatin, doxorubicin, which is a topoisomerase I inhibitor, or camptothecin, which is a topoisomerase II inhibitor, induced growth inhibition of #63 glioma cells at concentrations higher than 1 or 2.5 *μ*M, respectively. Each growth inhibition was also significantly enhanced in the presence of TRAIL as shown in Figures 3B and C. In addition, cell death induced by a combination of camptothecin and TRAIL showed clear changes in morphology that were typical of apoptotic cell death including decrease in cell size, condensed nucleus and formation of apoptotic bodies, as shown in Figure 3D. Moreover, DNA fragmentation induced by camptothecin in combination with TRAIL confirmed the apoptotic cell death as shown in Figure 3E.

### Treatment of #63 glioma cells with genotoxic agents induces the functional expression of TRAIL-R2

**Figure 4:**
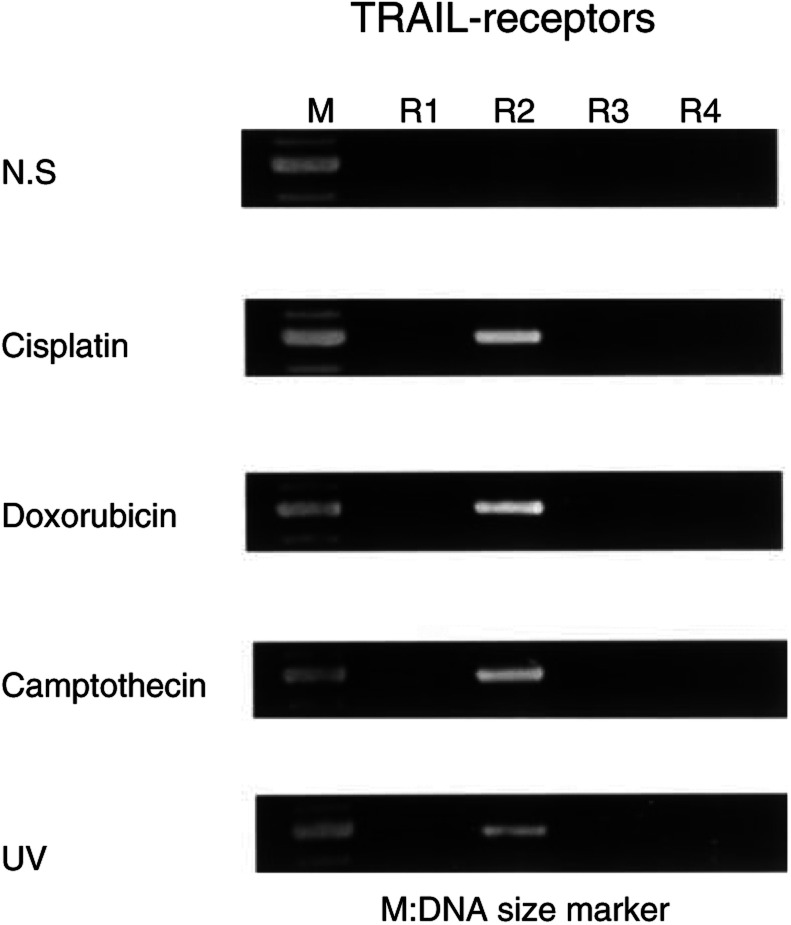
Specific induction of TRAIL-R2 in #63 cells by genotoxic agents. RT-PCR was performed using specific primers to each TRAIL receptor. Total RNA (5 *μ*g) was extracted from each cell culture with cisplatin (3 *μ*gml^−1^), doxorubicin (2.5 *μ*M) or camptothecin (2.5 *μ*M) for 24 h, or UV radiation (321 nm) for 5 min and another 3 h culture. Then reverse transcription and PCR amplification were performed. Each product was electrophoresed on 2% agarose gel and was visualised with ethidium bromide.

To explore the possibility that TRAIL-resistant cells restored the sensitivity of TRAIL-induced apoptosis, we first studied whether cisplatin could induce TRAIL receptor in #63 glioma cells that had not expressed TRAIL receptor at all. RT-PCR showed that the expression of TRAIL-R2 was specifically induced in #63 cells after treatment with cisplatin (3 *μ*gml^−1^) as shown in Figure 4

**Figure 5:**
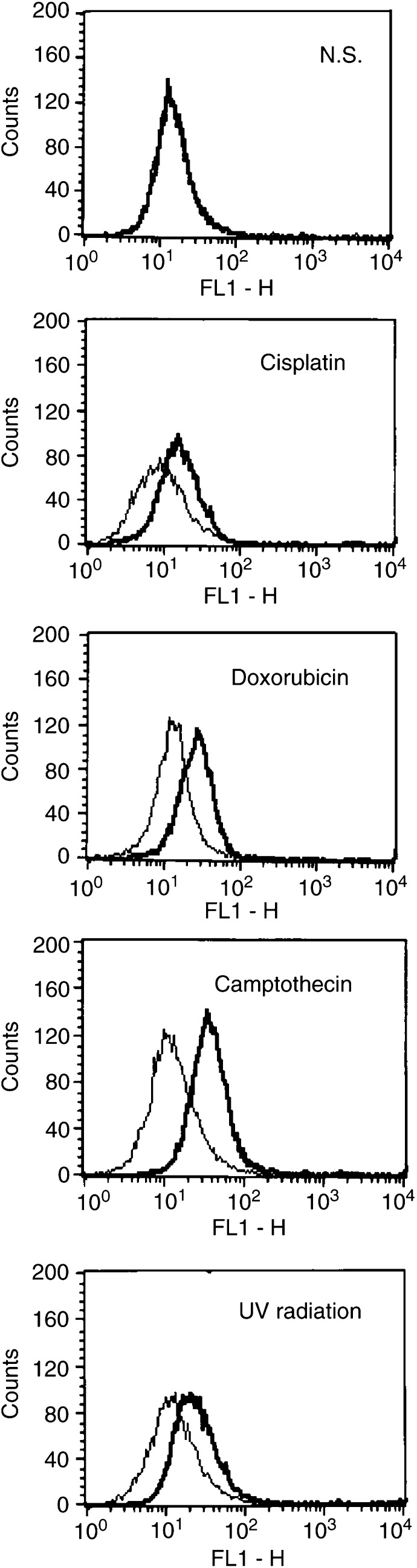
Cell surface TRAIL-R2 expression on #63 cells after treatment with genotoxic agents. After preculture with or without cisplatin (3 *μ*gml^−1^), doxorubicin (2.5 *μ*M) or camptothecin (2.5 *μ*M) for 24 h, or UV radiation (321 nm) for 5 min and another 8 h culture, cells were incubated with 10 *μ*gml^−1^ of anti-TRAIL-R2 Ab (thick line) or normal goat IgG (thin line) for 1 h. After washing with PBS, cells were incubated with FITC-conjugated secondary Ab. Then flow cytometric analysis was performed and fluorescence intensity was shown in a logarithmic scale.

**Figure 6:**
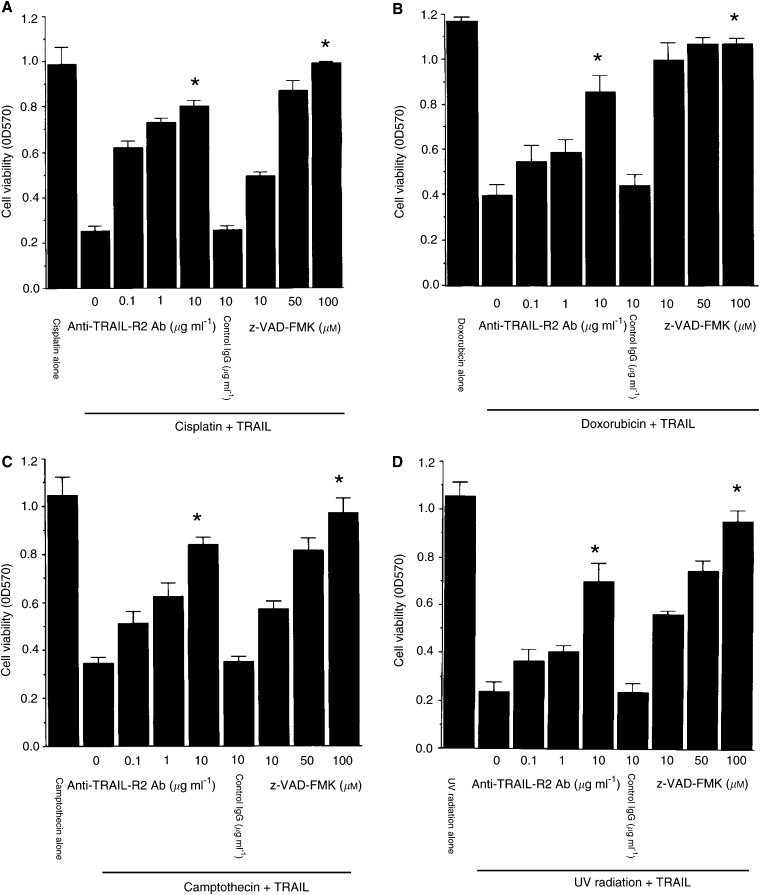
The effects of anti-TRAIL-R2 Ab or pan-caspase inhibitor on cell death after treatment of genotoxic agents and TRAIL. After preculture with genotoxic agents for 24 h, anti-TRAIL Ab or z-VAD-FMK in various concentrations was added 1 h before the addition of TRAIL. Then TRAIL (2 *μ*gml^−1^) was added to each culture and cell viability was measured by using crystal violet assay. The means and s.d.'s of five independent experiments are shown. Statistical significance: ^*^P<0.01 compared with controls.

. On the other hand, TRAIL-R1, -R3 and -R4 were not induced by cisplatin. Other genotoxic agents, such as doxorubicin or camptothecin, also induced the expression of TRAIL-R2 similarly to UV radiation, as shown in Figure 4. We next examined the cell surface TRAIL-R2 on #63 glioma cells by flow cytometric analysis. Cells were treated with anti-TRAIL-R2 Ab or isotype-matched control IgG and the cell surface expression of TRAIL-R2 on #63 cells was compared before and after treatment with genotoxic agents or UV radiation. As shown in Figure 5

, the cell surface expression of TRAIL-R2 was not detected on untreated #63 cells (top panel). However, it was induced by each stimulation, confirming the results obtained by RT-PCR. Lastly, we confirmed that the functional expression of TRAIL-R2 was the essential mechanism of sensitization of #63 cells to TRAIL by genotoxic agents using antagonistic anti-TRAIL-R2 Ab. As shown in Figures 6A–D

, cell death induced by cisplatin, doxorubicin, camptothecin or UV radiation in combination with TRAIL was effectively inhibited by neutralization of TRAIL-R2. In addition, cell death induced by combinations of genotoxic agents and TRAIL was inhibited by pan-caspase inhibitor z-VAD-FMK in a dose-dependent manner. These findings suggest that the functional cell surface expression of TRAIL-R2 on #63 glioma cells by genotoxic agents is the essential mechanism of sensitisation of #63 cells to TRAIL, and the subsequent death signal through TRAIL-R2 induces apoptosis of #63 cells via activation of caspases.

### DN-p53 inhibits the enhanced cytotoxity of TRAIL with genotoxic agents or UV radiation

**Figure 7:**
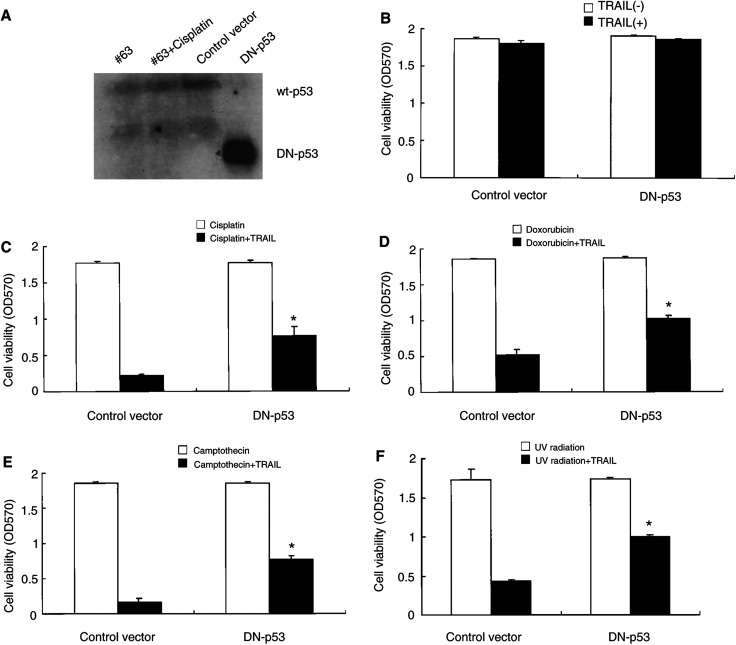
DN-p53 increases viability of #63 cells after treatment of TRAIL with genotoxic agents or UV radiation. The #63 cells were transfected with a DN-p53 construct or insertless construct (control vector). (**A**) Total proteins were extracted from #63 cells treated with or without 3 *μ*gml^−1^ cisplatin for 24 h and transfectants of either DN-p53 or control vector. Western blotting using specific Ab to C-terminal of wild-type p53 was performed and the specific bands were detected using ECL. Transfectants of DN-p53 or insertless construct (control vector) were treated for 24 h without (**B**) or with (**C**) 3 *μ*gml^−1^ cisplatin, 2.5 *μ*M doxorubicin (**D**), 2.5 *μ*M camptothecin (**E**), or UV radiation (**F**). Then TRAIL (2 *μ*gml^−1^) was added to each culture and cell viability was measured using crystal violet assay. The means and s.d.'s of five independent experiments are shown. Statistical significance: ^*^P<0.01 compared with controls.

An assay was developed to assess the importance of wild-type p53 on the induction of cell death of #63 cells. Cells were constitutively transfected with either a DN-p53 expression plasmid or an insertless control vector. To confirm the successful transfection of DN-p53 gene, we first performed Western blotting. As shown in Figure 7A

, DN-p53 was successfully expressed in transfected cells compared with transfectants of empty vector or the original #63 cells, which expressed wild-type p53. Each transfectant was cultured with each genotoxic agent or under UV radiation. As shown in Figures 7B–F, cell death was induced in a number of genotoxic agent-treated cells. In contrast, the viability was significantly increased in the DN-p53 transfected cells treated with genotoxic agents or UV radiation. These findings suggest that inhibition of the p53 function confers a survival advantage to #63 cells and the expression of TRAIL-R2 after exposure to genotoxic agents or UV radiation.

## Discussion

TRAIL/Apo2L is a member of the TNF family and can induce apoptosis in many tumour cells but not in normal cells. In fact, the present findings suggest that TRAIL induced apoptosis in eight of nine tumour cell lines, but not in normal osteoblasts or fibroblasts. These results are similar to those reported previously. Hao *et al*,(2001) showed that glioma cell lines were killed by TRAIL and normal astrocytes were resistant to TRAIL-induced cell death. Zhang *et al*,(2000) showed that human normal lung fibroblasts, melanocytes and umbilical vein endothelial cells were resistant to TRAIL-induced cell death even though they express cell surface TRAIL-R1 or -R2). Antitumour activity and safety of injection of TRAIL were also demonstrated *in vivo* (Ashkenazi *et al*, 1999a,1999b; Walczak *et al*, 1999). In contrast to Fas, which causes lethal liver failure (Tanaka *et al*, 1997), TRAIL is not toxic to normal cells, and thus is expected to be used for clinical use in cancer therapy. Recently, Kelley *et al*,(2001) reported no abnormalities associated with TRAIL injection in cynomolgus monkeys and chimpanzees and predicted characterizations of efficacy *in vivo*, pharmacokinetics and the safety of TRAIL in humans. Thus, cancer therapy using TRAIL is now expected clinically.

However, some tumour cells acquire resistance to TRAIL in various ways. Apoptosis mediated by TRAIL is regulated by the expression of two death receptors, TRAIL-R1 and TRAIL-R2, and two decoy receptors, DcR1 and DcR2, that compete with TRAIL-induced apoptosis (Degli-Esposti *et al*, 1997a; Marsters *et al*, 1997; Ashkenazi *et al*, 1999a). In previous studies, the expression of decoy receptors was not correlated with the sensitivity of TRAIL-induced apoptosis (Griffith *et al*, 1998; Hao *et al*, 2001), suggesting that the expression level of TRAIL receptors alone is not sufficient to account for the sensitivity to TRAIL-induced apoptosis. Several studies have suggested intracellular mechanisms for the TRAIL resistance of tumour cells. The expression level of FLIP was highest in the TRAIL-resistant melanoma cells and was low or absent in the sensitive cells, suggesting a correlation between TRAIL-induced apoptosis and the expression of FLIP (Griffith *et al*, 1998). TRAIL-resistant Ewing's sarcoma was shown to have low levels of caspase-8 both in mRNA and protein levels (Kontny *et al*, 2001). It has also been reported that PED/PEA-15 protein inhibits TRAIL-induced apoptosis of glioma cells (Hao *et al*, 2001), and cell lines overexpressing the erbB-2 receptor are resistant to TRAIL-mediated apoptosis (Cuello *et al*, 2001). Recently, mutations of TRAIL-R1 and -R2 were reported in breast cancers and these mutations were found more frequently in the metastatic tumours, suggesting that mutations in TRAIL-R1 and -R2 are possible mechanisms to escape TRAIL-induced apoptosis of tumour cells (Shin *et al*, 2001). In the present study, we first established a TRAIL-resistant cell line that completely lacked the expression of TRAIL receptors, which suggests one important mechanism for tumour cells to escape from TRAIL-induced apoptosis.

Some anticancer agents, in addition to UV radiation, have been shown to overcome the resistance to TRAIL, resulting in a synergistic effect with TRAIL. Wu *et al*,(1997) reported that TRAIL-R2 expression was enhanced after doxorubicin exposure only when wild-type p53 was present, but not in cells where it was mutated, degraded or not expressed. Sheikh *et al*,(1998) reported that ionizing radiation induced TRAIL-R2 in p53 wild-type cells, whereas methyl methane sulphonate regulated the expression of TRAIL-R2 in a p53-dependent and -independent manner, suggesting a cell-type-specific, trigger-dependent regulation of TRAIL-R2 expression. In contrast, Bachmann *et al*,(2001) suggested that TRAIL-R1 was downregulated upon UV exposure. In the present study, we confirmed the mechanisms of restoring the sensitivity by genotoxic agents or UV radiation to TRAIL-induced apoptosis, using transfection of DN-p53 in #63 cells. DN-p53 in #63 cells significantly inhibited the cytotoxity by TRAIL with genotoxic agents but not completely, suggesting that TRAIL-R2 expression by genotoxic agents or UV radiation at least partially depends on wild-type p53. Recently, p53-dependent upregulation of intra-cellular molecule Bak as in the case of Bax has been reported (LeBlanc *et al*, 2002). We observed the expressions of Bax and Bak in #63 cells, and it was upregulated by genotoxic agents (data not shown). Although our results using an antagonistic anti-TRAIL-R2 Ab support a potent mechanism of TRAIL resistance of #63 cells because of the lack of TRAIL receptors, the possibility that genotoxic agents may modify the signal transducers downstream of the receptors has to be elucidated. After all, these results suggest that combinations of anticancer agents and TRAIL might be the new strategies to overcome TRAIL resistance of tumour cells.

Recently, an immune response of TRAIL was reported. Type I IFN and anti-CD3 monoclonal antibody induce TRAIL expression on both CD4^+^ and CD8^+^ peripheral blood T cells (Kayagaki *et al*, 1999). Human CD11c^+^ blood dendritic cells express TRAIL after stimulation with either IFN-*γ* or -*α* (Fanger *et al*, 1999), and IFN-*β* upregulates TRAIL expression on human dendritic cells (Liu *et al*, 2001). IFN-*γ* or -*α* upregulates TRAIL expression on human monocytes (Griffith *et al*, 1999), and IL-12, an inducer of IFN-*γ* production, upregulates TRAIL expression on NK cells in the liver, spleen and lung (Smyth *et al*, 2001). In fact, TRAIL-deficient mice increased susceptibility to tumour initiation and metastasis, suggesting that TRAIL has an important role for immune surveillance by T cells, monocytes, dendric cells and NK cells (Cretney *et al*, 2002). In the present study, it is suggested that genotoxic agents not only directly kill tumour cells, but also induce apoptosis in tumour cells by utilizing an endogenously programmed cell-killing mechanism. These findings suggest the possible immune therapy for cancer using genotoxic agents by enhancing the susceptibility to TRAIL-bearing cytotoxic T, NK, NKT, dendritic cells and monocytes as well as administration of soluble TRAIL.
